# Record of strep throat infections in Italy: what is needed to know about penicillin allergy? The point of view from the Italian Society of Pediatric Allergy and Immunology (SIAIP)

**DOI:** 10.1186/s13052-023-01561-1

**Published:** 2024-02-14

**Authors:** Maria De Filippo, Angela Klain, Ilaria Brambilla, Silvia Caimmi, Riccardo Castagnoli, Cristiana Indolfi, Giulio Dinardo, Amelia Licari, Alberto Martelli, Maria Angela Tosca, Martina Votto, Gian Luigi Marseglia, Michele Miraglia del Giudice

**Affiliations:** 1 Department of Clinical, Surgical, Diagnostic and Pediatric Sciences, Pediatric Unit, University of Pavia, Pavia, Italy; 2 Pediatric Clinic, Fondazione IRCCS Policlinico San Matteo, Pavia, Italy; 3 Department of Woman, Child, and General and Specialized Surgery, University of Campania Luigi Vanvitelli, Naples, Italy; 4 Pediatric Unit, PO Garbagnate Milanese, ASST Rhodense, Milan, Italy; 5 Allergy Clinic, IRCCS Giannina Gaslini, Genoa, Italy

**Keywords:** Group A streptococcus, Invasive group A streptococcal (iGAS) infection, Pharyngitis, Amoxicillin, Drug hypersensitivity, Immediate reactions (IRs), Non-immediate reactions (NIRs)

## Abstract

Notifications of invasive group A streptococcal (iGAS) infections have significantly increased in many European Countries compared to the previous season. In Italy, there has been an increase in streptococcal pharyngitis and scarlet fever cases since January 2023, which sparked concerns about a GAS epidemic in the pediatric population. This rise may be ascribed to the GAS infection season that began earlier than usual (off-season outbreak) and the increase in the spread of respiratory viruses and viral coinfections that raised the risk of iGAS disease. Moreover, this phenomenon was also facilitated by increased travel after reduced GAS circulation during the COVID-19 pandemic.

The increase in cases of GAS disease has raised some critical issues regarding the potential reactions to administering amoxicillin, the first-line antibiotic therapy, many of which have been erroneously labeled as “allergy."

For these reasons, the Italian Society of Pediatric Allergy and Immunology (SIAIP) intends to provide simple clinical indications to help pediatricians manage GAS pharyngitis, discerning the allergic from non-allergic drug hypersensitivity.

To the Editor

group A streptococcal disease (GAS) ranges from mild symptoms to invasive or fatal disease [1].

In the second half of 2022, five European regions (France, Ireland, Netherlands, Sweden, and the United Kingdom) observed increased cases of invasive group A streptococcal disease (iGAS) and scarlet fever [2]. Moreover, some European Countries reported an increase in iGAS cases compared to the previous season, but with a lower incidence than before the COVID-19 pandemic [1]. GAS pharyngitis is frequent in winter and at the beginning of spring. Therefore, many authors call it an off-season outbreak.

According to European Centre for Disease Prevention and Control (ECDC) data, the most affected age groups are children younger than 10 years and people older than 65 years. Medical visits for scarlet fever and iGAS notifications peaked in the pre-Christmas period in December 2022 and then declined in January 2023. In Italy, an increase in scarlet fever cases was reported from January 2023 in children younger than 15 years [3]. The increase in scarlet fever cases concurrently occurred with an increased circulation of respiratory viral infections such as influenza and respiratory syncytial viruses, responsible for coinfections that increased the risk of iGAS disease. This phenomenon was encouraged by increased travel after reduced GAS circulation during the COVID-19 pandemic. Available data reported that iGAS cases are not linked to a specific/new strain or increased antibiotic resistance. For this reason, the World Health Organization (WHO) recommends promptly and correctly identifying and treating GAS-related infections, such as pharyngotonsillitis and scarlet fever, to reduce the risk of potential complications and subsequent transmission [1–3].

The primary clinical signs of streptococcal pharyngitis include fever, sore throat, and painful lymphadenitis [4]. Sometimes, the clinical picture evolves into scarlet fever, an exanthematous disease presented as a sandpaper-like confluent rash that involves the body and typically spares the perioral region. In the early stages, the tongue is covered with a whitish patina, in which the reddened papillae emerge on it (strawberry tongue); after 24–48 h, a widespread reddening with hypertrophic papillae (raspberry tongue) replaces the white patina on the tongue.

Basically, the diagnosis of GAS pharyngitis is based on the clinical condition, confirmed by the rapid detection of the bacterium from the pharyngeal swab (Rapid Strep Test, RST) [4]. However, the diagnosis is not always straightforward. It can be supportive of using the McIsaac score, which indicates the execution of the RST in selected patients, helping the clinician in the diagnostic of different etiologies. The score is based on the following criteria: patient’s age, body temperature, presence of tonsillar exudate, lymphadenopathy, and the absence of cough.

According to the score, a result below two requires no treatment or a confirmatory pharyngeal swab. A result between 2 and 4 indicates the execution of RST, and a result $\ge$ 5 indicates immediate antibiotic therapy. Evidence of a positive RST in the presence of a McIsaac score > 2 suggests antibiotic therapy. In the case of a McIsaac score > 2 with a negative RST but high diagnostic suspicion for GAS infection, it is necessary to perform a pharyngeal swab with culture and prescribe antibiotic therapy. There are some exceptions in which it is necessary recurring to the antibiotic therapy despite a low McIsaac score: 1) a child with positive RST and a family history of rheumatic disease, 2) a child with at least one symptom, a confirmed positivity of GAS infection in a familiar member, and a positive pharyngeal swab (Table 1) [4].

**Table 1:** McIsaac score

SIGNS/SYMPTOMS AND AGE	SCORE
Body temperature > 38°C	1
Absence of cough	1
Satellite lymphadenopathy	1
Exudate or increase in tonsil volume	1
Age 3–14	1
Age 15–44	0
Age > 45	-1

Antibiotic therapy remains an essential point of care for treating GAS infection. Antibiotic therapy should be prescribed within a few days of the symptom onset. The first-line drug is penicillin, the amoxicillin that should be administered orally at 50 mg/kg/day in 2–3 doses for 10 days. Alternatively, benzathine penicillin can be administered to a dosage of 600.000 IU in children with a weight < 27 kg and 1.200.000 IU in children with a weight ≥ 27 kg in a single intramuscular dose. In patients with a confirmed penicillin hypersensitivity but tolerance to cephalosporins, it is recommended the 2nd generation cephalosporin prescription, including cefaclor, cefprozil, cefuroxime axetil for 5 days, or macrolides (azithromycin 20 mg/kg/day once daily for 3 days or clarithromycin 15 mg/kg/day in 2 doses for 10 days) [4]. Unfortunately, the management of children with penicillin hypersensitivity is not as easy as it looks for our health system due to cephalosporins being quite costly and on the other hand there is a 20–40% resistance rate to macrolides. It is also important to emphasize, that in cases of severe reaction to penicillins, other beta-lactams such as cephalosporins cannot be prescribed without appropriate allergological work-up due to the risk of cross-reaction [5]. Moreover, despite the higher effectiveness, the percentage of severe adverse drug reactions (ADRs), including allergic ones, in many cephalosporin mixed preparations is greater than that in their respective individual components [6]. The sudden increase in cases of scarlet fever and streptococcal infection has led to a worldwide shortage of amoxicillin in a few months, and shortages of amoxicillin in pharmacies. Alternative antibiotics have been prescribed in pediatric age because of amoxicillin deficiency and because of misinterpretation of common skin rash during infection. Penicillin hypersensitivity is the most commonly reported drug allergy, with an estimated prevalence between 10–20% in adults and children, and upon thorough evaluation, it becomes 5–10%. Unlike adults, children are labeled as "penicillin allergic" because of the occurrence of a viral skin exanthema or non-allergic adverse events caused by the interaction between viral infection and antibiotic therapy (e.g., Ebstein-Barr virus (EBV) exanthema during the amoxicillin therapy) [7, 8]. Many viruses are directly implicated in exanthemas, such as EBV, herpesvirus 6 (HHV-6), cytomegalovirus (CMV), and so on [9]. Diagnosing skin eruptions during antibiotic treatment in the presence of a concurrent acute infection can be quite challenging for clinicians. Indeed, young children (particularly under 4 years of age) treated with penicillin, frequently develop an urticarial or maculopapular skin rash. According to the recent EAACI Task Force report, children with suspected mild to severe allergic reactions to penicillin should be referred to a pediatric allergologist [5]. The first step of the allergology management approach includes a detailed standardized history (data about symptoms presented, the specific morphology and parts of the body affected, the initial drug dose and duration, the time interval between drug administration and the reaction, treatment administered, and time to recovery, as well as previous episodes and drugs tolerated after the reaction) which allows in some cases to exclude a penicillin hypersensitivity or in the suspected cases of distinguished by immediate reactions (IRs), (occurring less than 1 h after the drug intake), and non-immediate reactions (NIRs), (occurring more than 1 h and up to several days after the last drug administration). In this regard, allergy tests should be performed 6–12 months after the hypersensitivity reaction. The allergy work-up usually consists of a skin test (ST), prick test, and if negative, intradermal tests (IDT) with penicillins followed by a drug provocation test (DPT). STs are usually associated with significant discomfort for children and parents; thus, many authors suggest reducing the number of STs or directly performing DPT with the suspected drug in children with NIRs mild reactions but the debate about children with IRs continues (Fig. 1) [10]. On the other hand, to address these issues current evidence proposes a new concept of “treating through” during acute skin mild non-immediate adverse drug reaction in patients who require a specific and not replaceable drug. This option is based on co-administration of H1-antihistamines and/or topical corticosteroids and may be a reasonable approach in selected patients, with careful risk–benefit assessment and under medical observation [11]. In conclusion, in this period of the GAS epidemic, the Italian Society of Pediatric Allergy and Immunology (SIAIP) Committee recommends referring children with suspected penicillin allergy to a pediatric allergy consultation before labeling them as “allergic” in order to prevent bacterial resistance and reduce the National Health System costs.

**Fig. 1:**
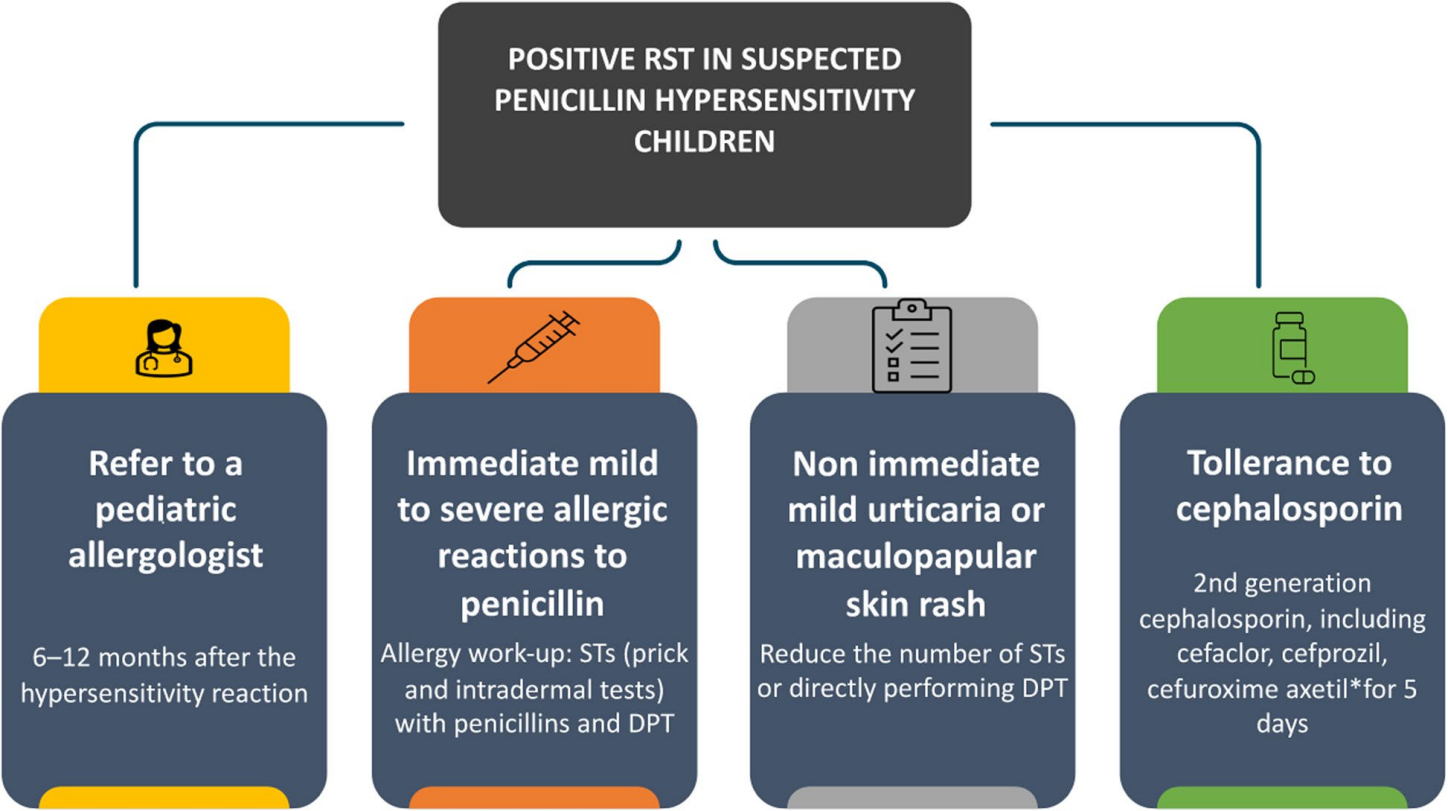
A practical approach to manage children with GAS infection and suspected penicillin hypersensitivity *The rate of cross-reactivity between penicillin and cephalosporin varied with the degree of similarity between R1 side chains, such risk is about 2.1% for cefuroxime axetil. RST: Rapid Strep Test, STs: Skin Tests STs, DPT: Drug Provocation Test

## Data Availability

Not applicable.

## Abbreviations

SIAIP: Society of Pediatric Allergy and Immunology
GAS: Group A streptococcal disease
iGAS: Invasive group A streptococcal disease
ECDC: European Centre for Disease Prevention and Control
WHO: World Health Organization
RST: Rapid Strep Test
EAACI: European Academy of Allergy & Clinical Immunology
ADRs: Adverse drug reactions
EBV: Ebstein-Barr virus
HHV6: Herpesvirus 6
CMV: Citomegalovirus
IRs: Immediate reactions
NIRs: Non-immediate reactions
ST: Skin test
IDR: Intradermal test
DPT: Drug provocation test
